# Orbital metastasis of renal cell carcinoma masquerading as thyroid ophthalmopathy

**DOI:** 10.1016/j.radcr.2024.12.052

**Published:** 2025-01-15

**Authors:** Smrti Aravind, Dhiran Sivasubramanian, Sathwik Sanil, Sharan Prasaanth, Virushnee Senthilkumar

**Affiliations:** a Coimbatore Medical College, Coimbatore, India; b Department of Critical Care Medicine, Christian Medical College, Vellore, India; c Institute of Oncology & Research, Sri Ramakrishna Hospital, Coimbatore, India

**Keywords:** Renal cell carcinoma, Orbital metastasis, Clear cell renal cell carcinoma, Thyroid ophthalmopathy, Magnetic resonance imaging (MRI), Palliative radiotherapy

## Abstract

Renal cell carcinoma (RCC) is the most common kidney malignancy in adults. However, its presentation with orbital metastasis as the first clinical manifestation is extremely rare and can mimic several other conditions. We report the case of a 72-year-old woman with a history of hyperthyroidism, who presented with symptoms resembling thyroid ophthalmopathy. However, magnetic resonance imaging (MRI) of the orbit revealed orbital metastasis. To find the primary tumor, a surveillance computed tomography (CT) was done which showed a mass in the left kidney suggestive of RCC. Histopathological analysis of the mass confirmed clear cell RCC. Early identification of orbital metastases in RCC is crucial, as it may indicate advanced disease. Misdiagnosis due to similarities with other orbital conditions can lead to delayed treatment and suboptimal outcomes. This case highlights the pivotal role of radiological imaging in guiding the diagnosis and management of orbital metastases from RCC.

## Introduction

Renal cell carcinoma (RCC) is a type of adenocarcinoma arising in the renal parenchyma [1]. RCC is the most common adult kidney cancer comprising 90% of cases [2]. Approximately 30% of RCC patients present with metastatic disease at diagnosis, and the most common metastatic sites include the lungs, bones, and liver [2]. However, orbital metastasis from RCC are extremely rare, accounting for less than 2% of all ophthalmic metastases [3]. These metastases can cause significant functional and cosmetic morbidity, often presenting as proptosis, diplopia, or pain, and are challenging to distinguish from other orbital conditions such as unilateral thyroid ophthalmopathy, idiopathic orbital inflammation, orbital arteriovenous malformations, cavernous hemangiomas, lacrimal gland tumors, and optic nerve tumors [[4], [5], [6]].

Clear cell renal cell carcinoma (ccRCC) is the most common histological subtype, accounting for 80% of all RCC cases followed by papillary RCC [7]. In contrast to papillary RCC, ccRCC is characterized by its hypervascularity, which facilitates hematogenous dissemination to highly vascularized sites, such as the lungs, thyroid, and even the orbit [8,9]. Orbital metastasis often correlates with advanced disease and a poor prognosis, reflecting the aggressive nature of ccRCC [10].

Here, we report a unique case of orbital metastasis in RCC mimicking unilateral thyroid ophthalmopathy. This case underscores the diagnostic challenges of distinguishing between pre-existing thyroid-associated orbital disease and rare metastatic lesions, highlighting the importance of comprehensive evaluation.

## Case presentation

A 72-year-old woman with a 10-month history of untreated hyperthyroidism, due to non-compliance, presented to the emergency department with chief complaints of right-eye proptosis and diplopia for the past 2 months. She reported progressive weight loss over recent months but denied any other comorbidities. There was no history of pain, discharge, or trauma associated with her symptoms.

Ten months earlier, the patient had visited a general practitioner with complaints of right eyelid edema and occasional palpitations. Routine blood investigations at that time showed elevated T3 and T4 levels with low TSH. She was prescribed antithyroid medications but did not adhere to the treatment.

The patient additionally reported intermittent lacrimation in the right eye and occasional flank pain. Physical examination revealed a right orbital swelling and a palpable left neck swelling. The best-corrected visual acuity was 6/9 in the left eye and 6/12 in the affected right eye. Ophthalmic examination revealed a firm, nontender mass over the right temporal part of the orbit with no skin changes or bruit. Right eye inferotemporal dystopia with 4-5 mm proptosis. Right eye abduction and elevation were restricted with diplopia on all gazes. Direct light reflex was sluggish in the right eye. Vitals on presentation included a respiratory rate of 18/min, heart rate of 102 beats/min, blood pressure of 120/70 mmHg, and a body temperature of 98.6°F. Examination of cardiovascular and pulmonary systems was unremarkable. Routine blood investigations, including liver and renal function tests, were within normal limits. Free T3 level was 8.80 pmol/L (reference range: 3.10–6.80 pmol/L), free T4 level was 24.28 pmol/L (reference range: 12.0–22.0 pmol/L), TSH was 0.08 mIU/L (reference range: 0.27–4.20 mIU/L).

**Fig. 1:**
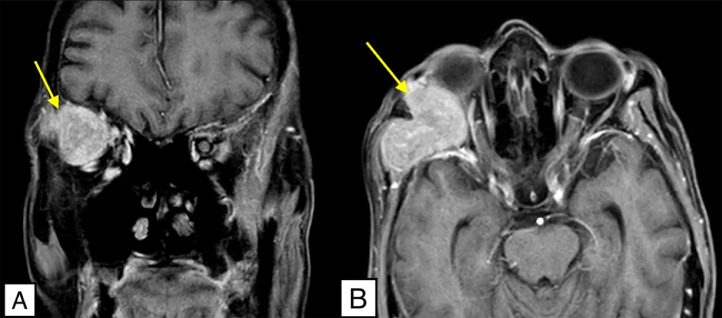
Contrast enhanced magnetic resonance imaging (MRI) of the orbit. (A) Coronal and (B) Axial T1 post-contrast images showing a hyperintense lesion with soft tissue component measuring 3.5 × 3 × 3.5 cm located in the right orbit (yellow arrow). It extends into intraconal region involving right temporalis muscle with lytic destruction of right greater wing of sphenoid and orbital process of frontal bone.

Initial differential included unilateral thyroid ophthalmopathy with a possibility of Graves' disease (6). Magnetic resonance imaging (MRI) of the orbit showed lytic destruction with enhancing soft tissue lesions involving the lateral wall of the right orbit, suggestive of orbital metastasis. It is seen involving the right temporalis muscle and also extending into intraconal region of the right orbit indenting the superior and lateral rectus muscles and the superior ophthalmic vein with mild deviation but no infiltration of the optic nerve (Fig. 1).

**Fig. 2:**
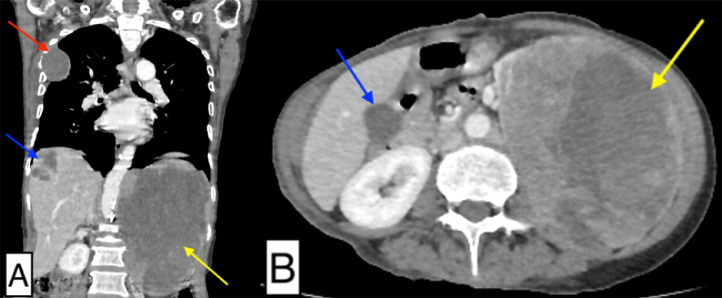
Contrast enhanced computed tomography (CT) of thorax and abdomen. (A) Coronal and (B) Axial images showing a large heterogeneously enhancing soft tissue mass lesion measuring 16 × 13.1 × 12.1 cm arising from the upper pole of the left kidney (yellow arrow). Well circumscribed lytic lesion measuring 6.6 × 4.5 × 4.7 cm arising from the lateral aspect of right 4th rib (red arrow). Multiple enhancing mass lesions in the liver parenchyma (blue arrow).

**Fig. 3:**
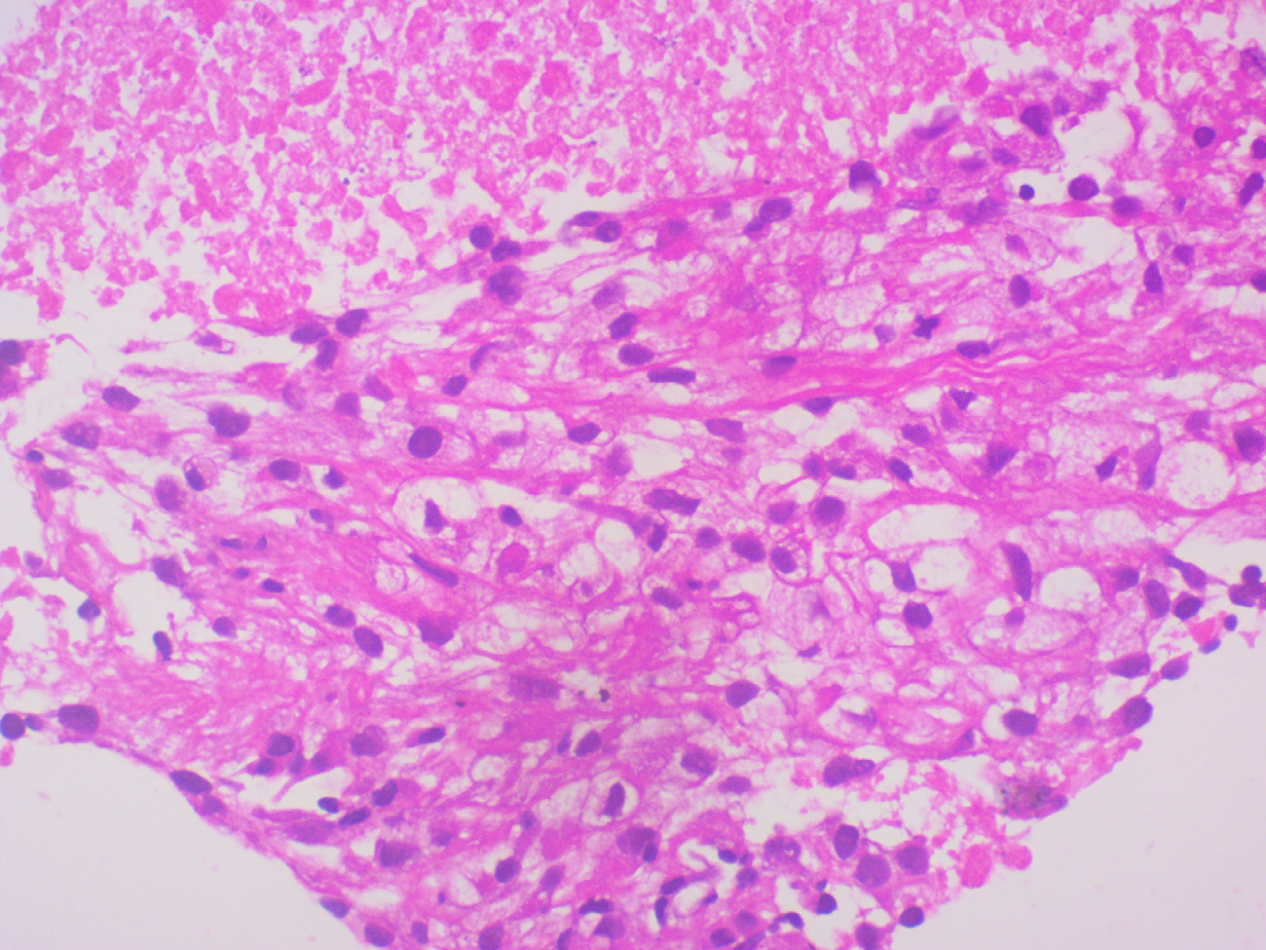
Histopathological examination of tru-cut biopsy specimen of the left kidney mass with hematoxylin and eosin staining showing tumor cells with abundant clear cytoplasm that are vacuolated with indistinct cell borders pathognomonic for clear cell renal cell carcinoma.

A surveillance computed tomography (CT) revealed a lytic mass lesion involving the upper pole of the left kidney with metastases to the liver, right 4th rib, left thyroid gland, and lungs (Fig. 2). To confirm the primary malignancy, an ultrasound-guided tru-cut biopsy of the mass in the left kidney was performed, histopathology confirmed the diagnosis of clear cell renal carcinoma (Fig. 3).

Due to financial constraints, the patient was unable to pursue surgical intervention or systemic therapies such as targeted treatments or immunotherapy. She was referred for palliative radiotherapy to address symptoms, particularly to alleviate orbital discomfort. The patient opted for hospice care, declining further treatments or diagnostic investigations.

## Discussion

Adult kidney cancers arise from either parenchyma or the collecting system. Cancers that arise from parenchyma are mainly adenocarcinomas, also known as renal cell carcinomas (RCC), while those that arise from the collecting system are mainly transitional cell carcinomas [1]. RCCs are the most common accounting for nearly 90% of the case [1]. Renal cell carcinomas are of 2 major histopathological subtypes: clear cell renal cell carcinoma (ccRCC) in 80% of the cases and papillary renal cell carcinoma in 15% of the cases [1,7,9]. ccRCC is a very vascular malignancy compared to papillary RCC, with a high potential to hematogenously spread, by direct extension into major vessels – the renal veins and the inferior vena cava [8,11]. They tend to spread to heavily vascularised structures such as lungs (45%), followed by bones (30%) and lymph nodes (22%) [2]. Orbital metastases are very rare, accounting for less than 2% of metastatic RCC cases, with ccRCC being the most frequent [4]. These lesions often mimic other orbital pathologies, delaying accurate diagnosis and treatment [5]. In one study, the interval between initial symptoms and diagnosis was as long as 11 months in some patients, emphasizing the need for heightened clinical suspicion when encountering atypical presentations of orbital diseases [12]. Only 6%-10% of RCC patients present with the classic triad of flank pain, gross hematuria, and a palpable abdominal mass, while most present with advanced disease and experience poorer outcomes. Imaging is the primary modality for diagnosing RCC, with the high accuracy of current imaging techniques, a biopsy is not typically required in cases of localised or locally advanced disease [13].

The patient's presentation with unilateral proptosis, diplopia, and a firm orbital mass initially suggested a thyroid etiology [14]. The most common cause of proptosis in adults is hyperthyroidism which typically presents with lid retraction, restrictive strabismus, and orbital edema [5,6]. However, atypical findings in this case, such as a palpable orbital mass with optic nerve impairment, prompted other possible etiologies. Metastatic RCC cases present with more varied and less specific symptoms depending on the metastatic site. This highlights the diagnostic complexity of RCC in its metastatic state.

This patient's systemic symptoms, including weight loss and flank pain, further emphasized the need for a thorough investigation. Imaging studies were crucial in distinguishing the underlying pathology. Orbital MRI revealed destructive and enhancing soft tissue lesions, findings that are inconsistent with benign thyroid-related conditions, but characteristic of metastatic disease [14]. Surveillance CT identified RCC as the primary malignancy, with metastases to multiple vascularized sites, including the liver, lungs, and thyroid gland. RCCs on CT are seen as solid with highly enhancing lesions or cystic masses with thick septa along with decreased attenuation. ccRCCs are visualized better with contrast-enhanced CT as they are highly vascularized [15].

The overlap between thyroid ophthalmopathy and orbital metastases from RCC exemplifies the need for meticulous evaluation in patients with atypical presentation. Orbital metastases are associated with high morbidity, and their presence often suggests a more aggressive and advanced disease course, with limited survival rates in untreated cases [3,10].

Due to financial constraints, the patient could not undergo curative surgery or systemic therapies such as tyrosine kinase inhibitors or immunotherapy. She received palliative radiotherapy, a standard approach for alleviating symptoms associated with orbital metastases, such as pain and diplopia [10]. While palliative radiotherapy does not significantly extend survival, it plays a critical role in improving quality of life.

This case underscores the importance of considering metastatic renal cell carcinoma (RCC) in the differential diagnosis of atypical orbital presentations, such as unilateral proptosis mimicking thyroid ophthalmopathy. Prompt imaging and thorough evaluation are essential for accurate diagnosis and timely management, especially in resource-limited settings. Early recognition of orbital metastases can guide appropriate palliative care, improving the quality of life in advanced RCC.

## Patient consent

Written informed consent for publication of this case was obtained from the patient.
